# Improved ASHCPWM with Reduced IGBT Switching Actions and Kalman Filter-Based Zero-Sequence Suppression for Open-End Winding PMSM Drives

**DOI:** 10.3390/mi17091081

**Published:** 2026-09-15

**Authors:** Yu Zhang, Mingzhe Qu, Hongjia Xie, Yang Xia, Liangxing Hu

**Affiliations:** 1 School of Intelligent and Architectural Engineering, Harbin University, Harbin 150086, China; qumingzhe@hrbu.edu.cn (M.Q.); xhjia@hrbu.edu.cn (H.X.); 2 College of Electrical Engineering, Harbin University of Science and Technology, Harbin 150080, China; 3 School of Electrical and Electronic Engineering, Nanyang Technological University, Singapore 639798, Singapore; yang_xia@ntu.edu.sg; 4 North Night Vision Technology Co., Ltd., Kunming 650217, China; 5 Kunming Institute of Physics, Kunming 650223, China

**Keywords:** IGBT inverter, reduced-switching PWM, ASHCPWM, zero-sequence current suppression, semiconductor loss

## Abstract

To improve the dc-bus voltage utilization while reducing the commutation burden of an insulated gate bipolar transistor (IGBT)-based open-end winding permanent magnet synchronous motor (OEW-PMSM) drive, this paper proposes an improved alternate sub-hexagonal center PWM (ASHCPWM) strategy. The drive employs two three-phase IGBT inverters sharing a common dc bus. Since the turn-off energy of an IGBT is strongly affected by its switching frequency, ASHCPWM alternately clamps one inverter and reduces the number of switching transitions. The resulting common-mode voltage mismatch, together with the dead-time voltage error required to prevent shoot-through in the IGBT bridge legs, nevertheless produces significant zero-sequence current and additional semiconductor current stress. A mathematical model of the zero-sequence voltage is therefore established, and a common-mode voltage compensation method is combined with an improved Kalman-filter-based harmonic extraction and closed-loop suppression strategy. The switching performance of the modulation is evaluated by counting the switching transitions of the twelve IGBTs and by comparing the zero-sequence current and phase-current spectra. Experiments on a 10 kHz prototype demonstrate that the proposed method substantially reduces zero-sequence harmonics while retaining the reduced-switching characteristic of the original ASHCPWM.

## 1. Introduction

The open-end winding (OEW) topology has received extensive attention in medium- and high-power motor-drive applications because it can achieve a higher output voltage than the conventional two-level inverter supplied by the same DC voltage source [1]. By using two three-phase inverters to drive the machine windings, the OEW topology can synthesize more voltage vectors and achieve multilevel output voltage, resulting in improved voltage utilization, lower harmonic distortion, enhanced fault-tolerant capability, and better power scalability [2]. Among various OEW configurations, the common DC-bus topology is attractive because of its simple structure, compact size, and low hardware cost. However, the shared DC bus forms an inherent zero-sequence current (ZSC) circulation path [3,4]. The common-mode voltage generated by the two inverters, together with the zero-sequence component of the back electromotive force (EMF) and the dead-time voltage error, excites the ZSC. As a result, the stator current distortion, copper loss, and torque ripple increase, leading to degraded drive performance [5]. Therefore, suppressing the ZSC while maintaining high voltage utilization is an important issue in common DC-bus OEW permanent magnet synchronous machine (OEW-PMSM) drives.

Compared with the series-end winding topology, the open-end winding topology provides higher control flexibility because the two ends of each phase winding are independently supplied by two inverters. Although the series-end winding topology can achieve a comparable linear modulation range with fewer inverter legs, the shared inverter legs may experience higher current stress. Moreover, the open-end winding topology provides a wider zero-sequence voltage regulation range, which is advantageous for active zero-sequence current suppression. Therefore, the open-end winding configuration is adopted in this work to facilitate the coordinated modulation and zero-sequence current control of the dual-inverter drive [6].

Many studies have investigated modulation strategies for ZSC suppression. A deadbeat predictive current control (DPCC) method combined with asymmetric hybrid carrier PWM (ASHCPWM) was proposed in [7], which suppresses the ZSC and torque ripple while maintaining full DC-bus voltage utilization. To improve the voltage synthesis capability, the ASHCPWM strategy was extended to the isolated DC-bus topology in [8]. The low-voltage inverter is clamped, while the high-voltage inverter synthesizes the remaining voltage vectors, thereby eliminating the unsynthesizable voltage region without increasing the switching frequency.

An improved implementation of ASHCPWM was further reported in [9], where the reference voltage vector is directly projected into the stationary ABC reference frame to calculate the switching time of each inverter phase. This method simplifies the implementation while preserving the modulation performance. Although these studies have improved voltage utilization and current quality, the modulation strategy is mainly developed from the viewpoint of voltage synthesis and current control. Silicon insulated-gate bipolar transistors (IGBTs) are still the preferred switching devices in many medium- and high-power motor drives because of their high voltage capability, mature manufacturing technology, competitive cost, and proven reliability [10]. However, excessive switching actions increase the switching loss, junction-temperature fluctuations, which can accelerate device aging and compromise the reliability of IGBT modules [11]. Therefore, reducing unnecessary switching transitions is beneficial not only for improving converter efficiency but also for enhancing the reliability of IGBT-based converters.

Silicon insulated-gate bipolar transistors (IGBTs) are still the preferred switching devices in many medium- and high-power motor drives because of their high voltage capability, mature manufacturing technology, and competitive cost. However, the turn-off tail current of IGBTs causes considerable switching loss, especially when the switching frequency increases. Excessive switching actions also increase junction-temperature fluctuation and reduce converter efficiency. Therefore, reducing unnecessary switching transitions is beneficial not only for improving converter efficiency but also for enhancing the reliability of IGBT-based converters. Although SiC MOSFETs and GaN devices provide lower switching loss and support higher switching frequencies, their higher cost and stricter gate-drive requirements limit their application in many industrial systems. Consequently, IGBT-based converters remain a practical choice in common DC-bus OEW drives. Recent studies have attempted to improve converter efficiency by optimizing the switching pattern. For example, multimode carrier-based PWM reduces the number of switching actions within one carrier period [12], while hybrid-inverter OEW systems employ IGBTs and MOSFETs simultaneously to exploit the advantages of both devices [13]. In common DC-bus OEW systems, ZSC suppression may also require increased switching frequency under certain operating conditions [14,15]. These results indicate that the modulation strategy should be designed by considering both current control performance and the switching characteristics of power semiconductor devices. However, the switching-transition characteristics of ASHCPWM have received limited attention in common DC-bus OEW drives.

In addition to modulation strategies, harmonic current suppression has also been widely investigated. Most existing methods consist of harmonic extraction followed by individual regulation of the extracted components. Among them, the multi-rotating reference frame (MRRF) method is widely adopted because of its simple implementation [16,17]. In this method, the harmonic components are transformed into their corresponding synchronous reference frames, and low-pass filters (LPFs) are employed to remove the interference from other harmonics. However, the LPF bandwidth directly affects the extraction performance. A narrow bandwidth improves the harmonic separation accuracy but slows the dynamic response, whereas a wider bandwidth accelerates the response at the expense of steady-state accuracy. This limitation becomes more evident at low speeds because the frequency separation between adjacent harmonics decreases. Although several improved extraction methods have been reported [18,19], the trade-off between dynamic response and steady-state accuracy still exists.

Based on the above analysis, the existing studies mainly focus on voltage synthesis, harmonic suppression, or switching-frequency reduction separately. The influence of IGBT switching characteristics on the modulation design has not been sufficiently considered, and the coordinated optimization of zero-sequence current suppression, harmonic reduction, voltage utilization, and switching actions has rarely been reported. To address these issues, this paper proposes an improved ASHCPWM strategy for common DC-bus OEW-PMSM drives. The proposed method suppresses the zero-sequence current and harmonic currents while reducing unnecessary switching transitions, thereby improving current quality and reducing unnecessary switching actions, which is beneficial for reducing switching-related losses and improving the overall drive performance.

On the basis of the above analysis, a new control method is developed in this paper. The main contributions are summarized as follows:

(1) The common-mode voltage characteristics of the ASHCPWM strategy are systematically analyzed, and a common-mode voltage compensation strategy is proposed to suppress the modulation-induced zero-sequence current. The proposed method improves the current quality while reducing unnecessary switching actions, which is beneficial for alleviating the switching stress of the IGBT-based inverter.
(2) An improved Kalman filter is proposed to separate and extract the fundamental and harmonic current components, enabling accurate harmonic identification and providing reliable current information for harmonic compensation.
(3) Harmonic-specific mathematical models are established, and the Kalman filter parameters are designed to suppress the mutual interference among different harmonic components. As a result, the proposed method achieves a good balance between dynamic response and steady-state accuracy while maintaining the modulation performance.

The remainder of this paper is organized as follows. Section 2 analyzes the conventional ASHCPWM strategy and establishes the mathematical model of the common-mode voltage. Section 3 presents the proposed common-mode voltage compensation strategy, the improved Kalman filter-based harmonic extraction method, and the corresponding harmonic current control scheme. Section 4 provides the experimental results to verify the effectiveness of the proposed method in suppressing the zero-sequence current, improving harmonic current compensation, and maintaining the reduced-switching characteristic of ASHCPWM. Section 5 concludes this paper.

## 2. IGBT-Based Dual-Inverter ASHCPWM

**Figure 1:**
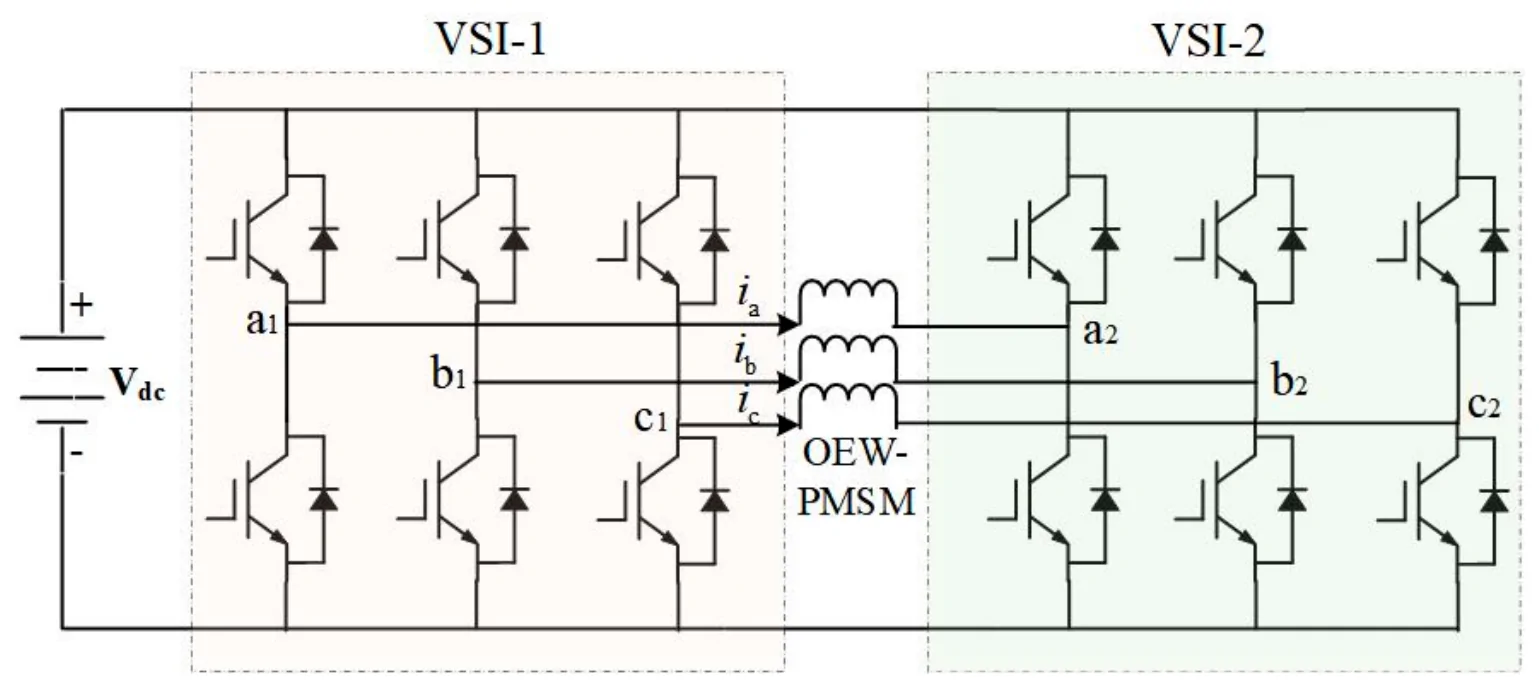
Open-end winding topology.

The motor drive topology adopted in this paper consists of a DC power supply and two three-phase inverters sharing a common DC bus, where each phase leg of the inverter is composed of two IGBTs. The two terminals of each phase winding of the open-end winding PMSM are connected to the midpoints of the corresponding phase legs of the inverters on both sides, and the difference between the voltage vectors generated by the two inverters constitutes the voltage vector applied to the PMSM, as illustrated in Figure 1. VSI-1 and VSI-2 denote the two voltage source inverters. *i*_a_, *i*_b_, *i*_c_ represent the three-phase winding currents, and a_1_, b_1_, c_1_, a_2_, b_2_, c_2_, are the output terminals of VSI-1 and VSI-2, respectively.

In conventional drive schemes, the two inverters generate voltage vectors with identical magnitudes and a phase displacement of 180° or 120°, which requires the IGBTs of both inverters to switch once within each control period, resulting in a relatively large number of switching actions and a high effective switching frequency of the IGBTs.

### 2.1. Conventional ASHCPWM

**Figure 2:**
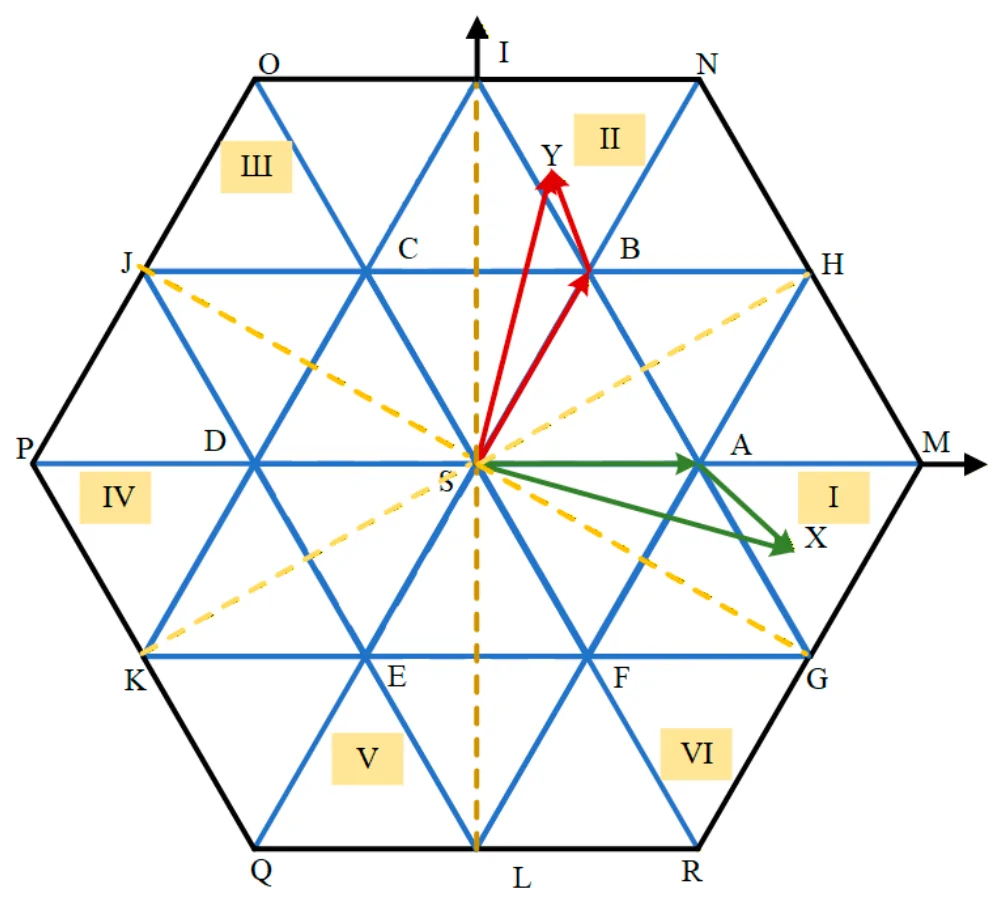
ASHCPWM vector synthesis method.

Compared with a conventional three-phase inverter, the open-end winding topology employs twelve switches, providing higher control flexibility and a wider voltage modulation range. To reduce switching losses, ASHCPWM fixes the switching state of one inverter during each control period, while the other inverter synthesizes the desired voltage vector using SVPWM. Taking Section I in Figure 2 as an example, Inverter I is clamped at (1-0-0) to generate a fixed voltage vector, and Inverter II produces the remaining voltage vector component via SVPWM.

Unlike conventional single-inverter SVPWMs, whose voltage vectors are limited to the inscribed circle of the hexagon, ASHCPWM allows the synthesizable voltage vectors to cover the entire hexagonal region in each section shown in Figure 2. Since one inverter remains in a fixed switching state within a control cycle, the number of switching transitions is reduced, and the effective switching frequency of the power devices can theoretically be reduced by approximately half compared with the conventional method.

However, the reduced-switching characteristic of conventional ASHCPWMs is achieved at the expense of unequal common-mode voltages generated by the two inverters. When one inverter is clamped at a fixed switching state while the other inverter performs PWM modulation, the common-mode voltages on the two sides generally cannot remain identical, resulting in a zero-sequence voltage across the motor windings and consequently exciting zero-sequence current. Therefore, the objective of the improved ASHCPWM proposed in this paper is to retain the reduced-switching characteristic of the conventional ASHCPWM while compensating for the modulation-induced common-mode voltage mismatch. In this way, the zero-sequence current can be effectively suppressed without requiring both inverters to perform continuous PWM switching.

### 2.2. Common-Mode Voltage

The range of achievable rotating voltage vectors is shown in Figure 2. However, the voltage vector synthesis analysis in the rotating voltage plane does not consider the zero-sequence voltage component, potentially resulting in significant zero-sequence currents.

Phase terminal voltages of inverter I can be defined as *v*_a1_, *v*_b1_ and *v*_c1_. Phase terminal voltages of inverter II can be defined as *v*_a2_, *v*_b2_ and *v*_c2_. The actual phase voltages applied across the motor windings can be calculated according to:

(1) $$v_{a}=v_{a1}-v_{a2}$$

(2) $$v_{b}=v_{b1}-v_{b2}$$

(3) $$v_{c}=v_{c1}-v_{c2}$$

The common-mode voltage of each inverter can be defined as:

(4) $$v_{cm1}=\frac{v_{a1}+v_{b1}+v_{c1}}{3}$$

(5) $$v_{cm2}=\frac{v_{a2}+v_{b2}+v_{c2}}{3}$$

Therefore, the zero-sequence voltage applied to the stator is:

(6) $$v_{0}=\frac{v_{a}+v_{b}+v_{c}}{3}=v_{cm1}-v_{cm2}$$

When the conventional ASHCPWM method is applied, within one control period, one inverter maintains fixed switching states, such as (1-0-0) or (1-1-0), resulting in a corresponding common-mode voltage of *U*_dc_/3 or 2*U*_dc_/3. The other inverter modulates the voltage vectors using the SVPWM scheme, leading to a time-varying common-mode voltage. The mismatch between the common-mode voltages of the two inverters gives rise to zero-sequence currents.

Taking the zero-voltage vector *S* as an example, this voltage vector can be synthesized by the SA vector generated by Inverter I and the SA vector generated by Inverter II. This means that, within one control cycle, both Inverters I and II will maintain a fixed switching state. The active voltage vector occupies the entire control cycle, with no remaining time for zero-sequence vectors. In this case, the open-end winding topology cannot regulate to control zero-sequence components. For Section I, the closer the voltage vector is to S or the HMG boundary, the greater the proportion of the duty cycle that Inverter II must allocate to active voltage vectors for active voltage vectors. Consequently, the range of achievable zero-sequence voltage becomes smaller, and the ability to regulate zero-sequence current becomes weaker.

Within one switching period *T*_s_ the modulation uses two adjacent active vectors and the remaining time for zero vector. Let *T*_1_ = time allocated to the “two-high, one-low” active vector (e.g., 110), *T*_2_ = time allocated to the adjacent “one high, two-low” active vector (e.g.,100), *T*_z_ = *T*_s_ − (*T*_1_ + *T*_2_) = total zero-vector time, and a fraction α ∈ [0, 1] of *T*_z_ is assigned to (111) (all high) and the remaining 1 − α to (000) (all low).

For a “two-high, one-low” state:

(7) $$V_{cm}^{\left(2h1l\right)}=\frac{2U_{\mathrm{dc}}}{3}$$

For a “one-high, two-low” state:

(8) $$V_{cm}^{\left(1h2l\right)}=\frac{U_{\mathrm{dc}}}{3}$$

For zero states:

(9) $$V_{cm}^{\left(111\right)}=U_{\mathrm{dc}}$$

(10) $$V_{cm}^{\left(000\right)}=0$$

The period average (weighted by durations) is

(11) $$V_{cm}(\alpha )=\frac{U_{\mathrm{dc}}}{T_{s}}(\frac{2}{3}T_{1}+\frac{1}{3}T_{2}+\alpha ,T_{z})$$

This expression shows that, for fixed *T*_1_, *T*_2_, *T*_z_, changing α shifts *V*_cm_ linearly between two extremes.

If all zero time goes to (0-0-0) (α = 0), the minimum common-mode:

(12) $$V_{cm}^{\min}=\frac{U_{\mathrm{dc}}}{T_{s}}(\frac{2}{3}T_{1}+\frac{1}{3}T_{2})$$

If all zero time goes to (111) (α = 1), the maximum common-mode:

(13) $$V_{cm}^{\max}=\frac{U_{\mathrm{dc}}}{T_{s}}(\frac{2}{3}T_{1}+\frac{1}{3}T_{2}+T_{z})$$

Using *T*_z_ = *T*_s_ − (*T*_1_ + *T*_2_), these can be rewritten as:

(14) $$V_{cm}^{\min}=\frac{U_{\mathrm{dc}}}{T_{s}}(\frac{2}{3}T_{1}+\frac{1}{3}T_{2})$$

(15) $$V_{cm}^{\max}=\frac{U_{\mathrm{dc}}}{T_{s}}(T_{s}-\frac{1}{3}T_{1}-\frac{2}{3}T_{2})$$

The width of the achievable *V_cm_* interval (difference between max and min) equals the contribution of the zero-vector time:

(16) $$V_{cm}^{\max}-V_{cm}^{\min}=\frac{U_{\mathrm{dc}}}{T_{s}}T_{z}$$

Thus, assigning the zero time between (000) and (111) can move *V*_cm_ across an interval of length *U*_dc_, *T*_z_/*T*_s_.

Because the basic active states only produce *V_cm_* of 2 *U*_dc_ /3 or *U*_dc_ /3, the zero-vector allocation (between 0 and *U*_dc_) dominates the long-term common-mode bias when *T*_z_ is large. By choosing α you can place the average *V_cm_* anywhere between the two extremes. If *T*_1_ and *T*_2_ change (as the reference vector angle changes), the whole interval [$V_{cm}^{\min}$,$V_{cm}^{\max}$] shifts accordingly.

## 3. Zero-Sequence Current Suppression

Application of ASHCPWM to motor drives results in multiple harmonic currents. The sources of these harmonic currents include the common-mode voltage induced by the modulation method and the voltage distortion caused by the dead-time effect. To suppress these harmonic currents, especially the zero-sequence harmonics, it is necessary to extract these harmonics from the three-phase currents and independently regulate them through closed-loop control.

### 3.1. Conventional Harmonic Extraction

In addition to the main positive-sequence fundamental-frequency current, the three-phase currents include negative sequence 5th-harmonic current, positive-sequence 7th-harmonic current, etc., caused by the dead-time effect, as well as zero-sequence currents of multiples of 3 induced by the common-mode voltage. For rotating current vectors, rotating coordinate systems can be established separately to transform the three-phase currents into different coordinate systems. Within a rotating coordinate system of a specific frequency, the rotating current vector at that frequency behaves as a direct current (DC), while harmonic components of other frequencies appear as alternating harmonic components. Therefore, low-pass filters (LPFs) can be employed to extract each harmonic while suppressing interference from other harmonics. However, it should be noted that the performance of LPFs is not ideal. Although LPFs can attenuate the amplitude of harmonics, they cannot completely eliminate interference, which inevitably results in residual interference in the extracted harmonics.

In addition, the primary drawback of the multiple rotating coordinate system (MRCS) method in harmonic extraction is its inability to extract zero-sequence harmonics. Rotating current vectors are two-dimensional, enabling the establishment of two-dimensional coordinate systems rotating at the same frequency for harmonic extraction. However, zero-sequence currents are one-dimensional, and it is not feasible to construct corresponding coordinate systems for zero-sequence harmonic currents at different frequencies of different frequencies. Consequently, harmonic extraction cannot be achieved for these components.

### 3.2. Harmonic Extraction Based on Kalman Filter

The adopted modulation method results in zero-sequence current harmonics of order 3n, with the 3rd harmonic being the dominant component. In addition to introducing a small amount of zero-sequence harmonics, the deadtime mainly induces negative-sequence 5th-order, positive-sequence 7th-order and other harmonics. For simplicity, when modeling the open-end winding PMSM, we first consider only the positive-sequence fundamental frequency component, negative-sequence 5th-order harmonic and zero-sequence 3rd-order harmonic, which can be expressed as:

(17) $$v^{+1}=V^{+1}e^{j\theta e}$$

(18) $$v^{-5}=V^{-5}e^{-j5\theta e}$$

(19) $$v^{3}=V^{3}\sin(5\theta_{e}+\varepsilon )$$

where θe denotes the rotor position. The superscripts “+1” and “−5” represent the positive fundamental frequency and negative sequence 5th-order, respectively. The superscript “3” represents zero-sequence 3rd-order.

The positive and negative sequence components are decomposed into their real and imaginary parts, corresponding to the α- and β-axis components in the stationary reference frame. The mathematical model of the positive and negative sequence components can be expressed as

(20) $$x_{k+1}=F_{k}x_{k}+w_{K}$$

where

(21) $$x_{k}={\left[{ℜ}_{k}^{+}\;{ℑ}_{k}^{+}\;{ℜ}_{k}^{-}\;{ℑ}_{k}^{-}\;{ℜ}_{k}^{\left(3,0\right)}\;{ℑ}_{k}^{\left(3,0\right)}\right]}^{\Gamma }$$

(22) $$F_{k}=\left[\begin{array}{ccc} R\left(\phi_{k}\right) & 0 & 0\\ 0 & R\left(-5\phi_{k}\right) & 0\\ 0 & 0 & R\left(3\phi_{k}\right) \end{array}\right]$$

(23) $$R\left(\phi \right)=\left[\begin{array}{cc} \cos\phi & -\sin\phi \\ \sin\phi & \cos\phi \end{array}\right]$$

Here, $\phi_{k}=\theta_{k}-\theta_{k-1}$ denotes the grid phase angle difference between two control periods, and *w*_k_ represents the process noise, defined as

(24) $$w_{k}={\left[w_{1}^{+1}\;w_{2}^{+1}\;w_{1}^{-5}\;w_{2}^{-5}\;w_{1}^{3}\;w_{2}^{3}\right]}^{T}$$

In the traditional Kalman filter, the process noise is assumed to follow a zero-mean normal distribution *w*_k_ ∼ *N*(0, Q). Since the positive and negative sequence components strictly follow the above dynamic model, the process noise is assumed to be zero, i.e., Cov(*w*_k_) = 0.

The observation equation can be expressed as:

(25) $$y_{k}=\left[\begin{array}{c} v_{\alpha ,k}\\ v_{\beta ,k}\\ v_{0,k} \end{array}\right]=H_{Xk}+vk$$

where

(26) $$y_{k}=\left[\begin{array}{cccccc} 1 & 0 & 1 & 0 & 0 & 0\\ 0 & 1 & 0 & 1 & 0 & 0\\ 0 & 0 & 0 & 0 & 1 & 0 \end{array}\right]$$

*v*_k_ denotes the measurement noise. Since the Kalman filter model considers only the fundamental positive sequence fundamental frequency, negative sequence 5th-order components and zero sequence 3rd-order components while neglecting other harmonic disturbances, the measurement noise term v is introduced, and its covariance is given by Cov(*v*_k_) = *R*. It should be noted that, in the Fmodel, the zero-sequence component is decomposed into its real and imaginary parts. However, only the real part is incorporated into the output matrix *H*. Consequently, the observed real part of the zero-sequence component corresponds to the actual zero-sequence current, whereas the imaginary part represents a $90^{\circ }$ phase-shifted version of the real part. Although the imaginary part has no direct physical meaning in the model, it can be utilized to facilitate zero-sequence current regulation in the subsequent closed-loop control. With the availability of the quadrature signal, the zero-sequence third-order harmonic can be transformed into a dc component via the Park transformation, regulated using a conventional PI controller, and then inversely transformed back to the third-harmonic reference frame, thereby enabling accurate closed-loop control of the third-order harmonic.

Based on the above linear model of the positive and negative sequence components, a standard Kalman filter algorithm is adopted. The Kalman filter is first initialized with the state variables and the a priori covariance matrix *P*. The prediction step is given by

(27) $$\left\{\begin{array}{l} {\hat{X}}_{k|k-1}=F,{\hat{X}}_{k-1|k-1}\\ P_{k|k-1}=FP_{k-1|k-1}F^{T} \end{array}\right.$$

It should be noted that, since the system model is assumed to be accurate, the process noise covariance *Q* is not included in the computation of *P*_k|k−1_.

The update step is expressed as:

(28) $$\left\{\begin{array}{l} K_{k}=P_{k|k-1}H^{T}{\left(HP_{k|k-1}H^{T}+R\right)}^{-1}\\ {\hat{X}}_{k|k}={\hat{X}}_{k|k-1}+K_{k}\left(y_{k}-H{\hat{x}}_{k|k-1}\right)\\ P_{k|k}=\left(I-K_{k}H\right)P_{k|k-1} \end{array}\right.$$

This procedure is identical to that of the conventional Kalman filter. Although the proposed Kalman filter can accurately extract the positive and negative sequence components, its convergence becomes slow when the amplitudes of these components vary after the filter has reached steady state. Therefore, an adaptive adjustment of the Kalman filter parameters is considered to achieve a trade-off between dynamic response speed and steady-state accuracy.

### 3.3. Improved Kalman Filter

The Kalman filter presented in the foregoing sections can achieve a high dynamic response speed when the a priori error *P*_k|k−1_ is large. However, it has a serious drawback: after the initial observation converges, the Kalman filter responds slowly to subsequent harmonic variations. This is because the convergence of the initial observation causes the a posteriori error *P*_k|k_ to converge to 0. An excessively small *P*_k|k_ renders the algorithm unable to achieve a rapid response to subsequent harmonic variations, and it has to wait until *P*_k|k_ increases to restore its fast tracking capability.

To prevent the Kalman filter from losing its fast response capability after observation convergence, it is necessary to dynamically adjust the a priori error *P*_k|k−1_ during algorithm operation. The method adopted in this paper is to set an observation error threshold: when the observation error exceeds this threshold, *P*_k|k−1_ is no longer set based on the a posteriori error *P*_k|k_ calculated in the previous control cycle, but is instead continuously increased. The increased *P*_k|k−1_ drives the Kalman filter to achieve fast convergence, reduces the observation error to within the threshold, allows *P*_k|k−1_ to reconverge to 0, and thereby realizes the convergence of the observation.

Compared with the conventional harmonic extraction method based on rotating reference frames and LPFs, the proposed Kalman-filter-based method does not rely on the LPF bandwidth to separate different harmonic components. Therefore, it avoids the inherent trade-off between dynamic response and steady-state filtering accuracy associated with LPF-based methods. In addition, the adaptive adjustment of the error covariance enables the proposed method to recover a high tracking capability when abrupt harmonic variations occur, while maintaining accurate harmonic separation under steady-state conditions.

**Figure 3:**
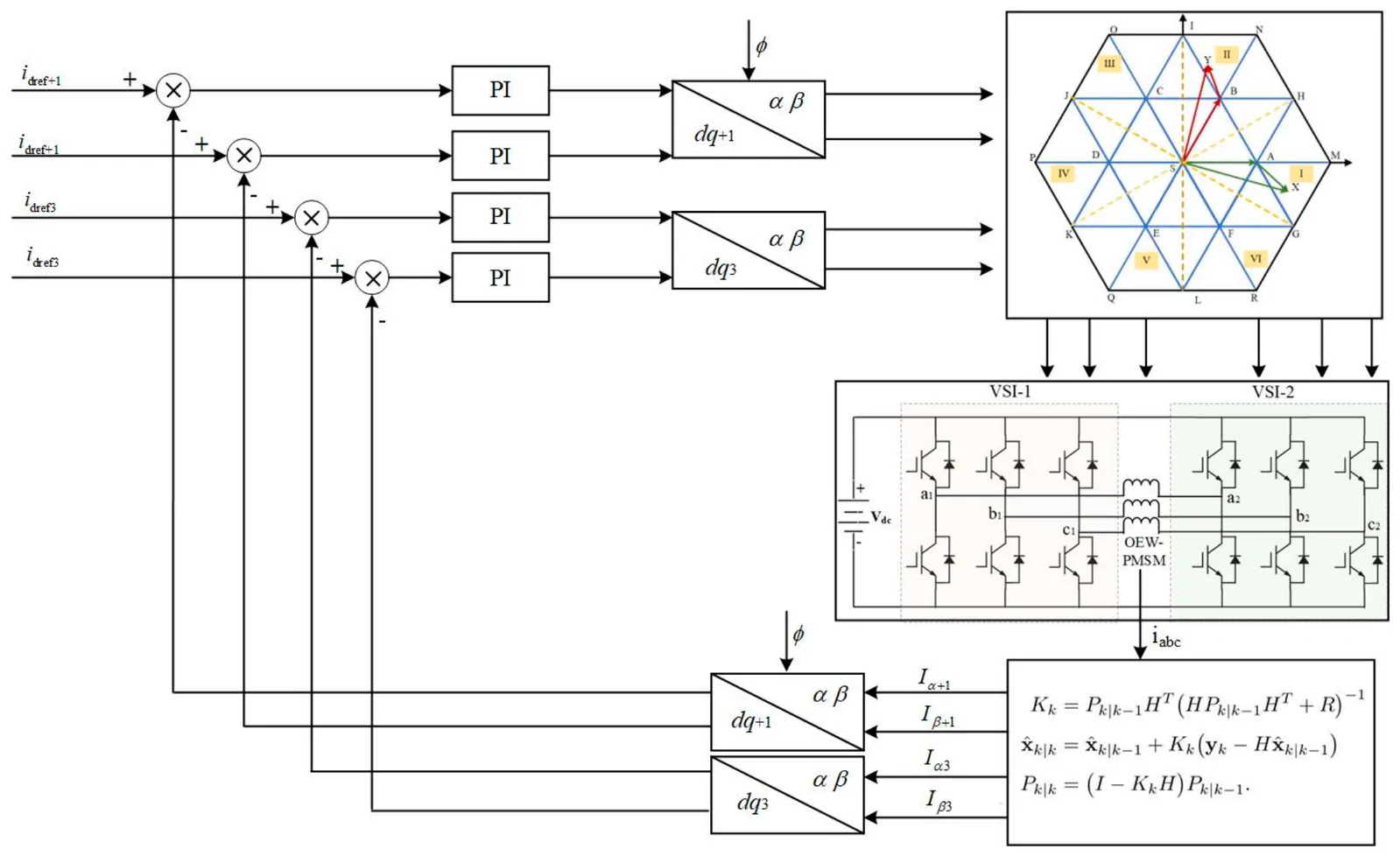
Overall structure of the controller.

The overall control principle block diagram is illustrated in Figure 3. The improved Kalman filter extracts the positive-sequence fundamental current, the zero-sequence third-order harmonic current, and the corresponding 90 phase-shilled signal of the third-order harmonic from the three-phase currents. Through the Park transformation, both the positive-sequence fundamental current and this set of third-order harmonic components are converted into dc quantities. The voltage commands generated by the PI controllers in the rotating reference frames are transformed via the fundamental-frequency and triple-frequency rotating transformations to obtain the final voltage references applied to the ASHCPWM module, which subsequently generates the gate-driving signals for the six switching bridge legs. It should be noted that only the real part of the third-order harmonic voltage reference is injected as the zero-sequence component, while the imaginary part is not utilized.

## 4. Experimental Results

### 4.1. Analysis of EMF Modulation Comparison

To verify the effectiveness of the proposed modulation strategy, the OEW-PMSM is evaluated under three control schemes: conventional dual-SVPWM, ASHCPWM without zero-sequence compensation, and the proposed ASHCPWM with zero-sequence compensation. A constant-magnitude rotating voltage vector is applied, and the resulting six-phase current waveforms and harmonic spectra are compared to assess the suppression of zero-sequence components.

**Figure 4:**
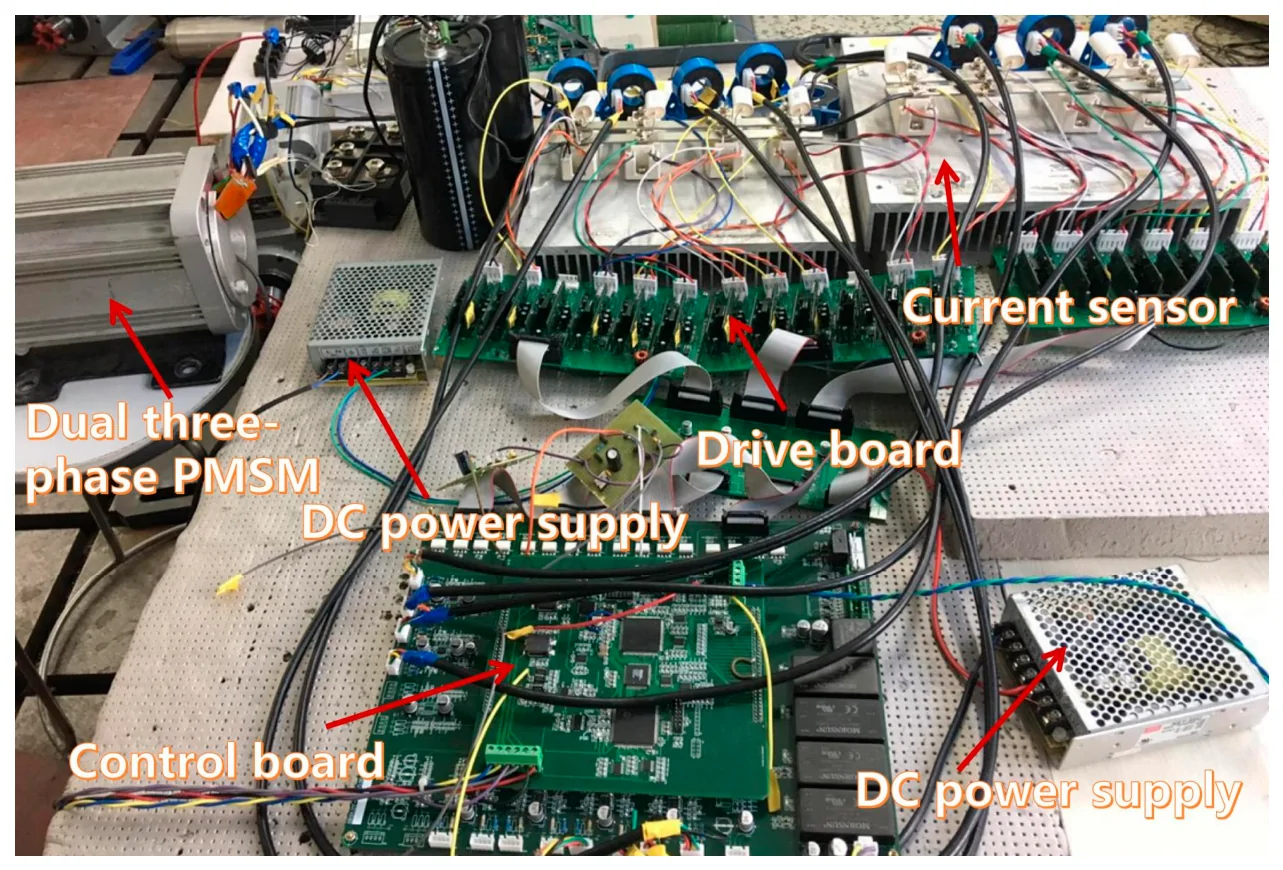
Experiment platform based on dual-three-phase PMSM and inverter.

An experimental platform is established to validate the proposed control strategy for the six-phase PMSM, as shown in Figure 4. The test bench consists of a six-phase PMSM, a magnetic powder brake, a three-phase diode rectifier, a DC-link capacitor bank, a six-phase IGBT voltage-source inverter, a digital control board, gate driver circuits, current and voltage sensing units, and auxiliary power supplies. The magnetic powder brake is mechanically coupled to the PMSM shaft and is used as the mechanical load during the experiments. The main parameters of the experimental PMSM are listed in Table 1. The power stage employs an MDS150D-16 diode rectifier to provide the DC-link voltage, while the DC-link is formed by two 3300 μF/400 V electrolytic capacitors connected in series. The inverter is implemented using GD50HFU120C1S IGBT modules rated at 1200 V and 50 A and is driven by 2SD315A gate-driver modules. The switching frequency is set to 10 kHz.

In the experiments, the electrical fundamental frequency is set to 10 Hz. It should be noted that the objective of the experiments is to evaluate the proposed modulation and harmonic suppression methods rather than the rated operating performance of the motor. A relatively low fundamental frequency is intentionally selected so that the fundamental component and the dominant low-order harmonics, particularly the 3rd- and 5th-order harmonics, can be clearly distinguished in the frequency spectrum. Accordingly, the 3rd- and 5th-order components appear at 30 Hz and 50 Hz, respectively, which facilitates the quantitative comparison of the harmonic suppression performance. In addition, the dead-time-induced current distortion is more readily observable around the current zero-crossing points at low-frequency operation, providing a clear condition for experimentally evaluating the proposed compensation method.

To further experimentally evaluate the power performance of the converter system, the input power is monitored directly from the display of the DC power supply under the same operating conditions. The experimental results demonstrate that the proposed modulation strategy reduces the input power of the converter system from approximately 510 W to 495 W under the same operating condition, while effectively suppressing the dominant low-order harmonics.

**Figure 5:**
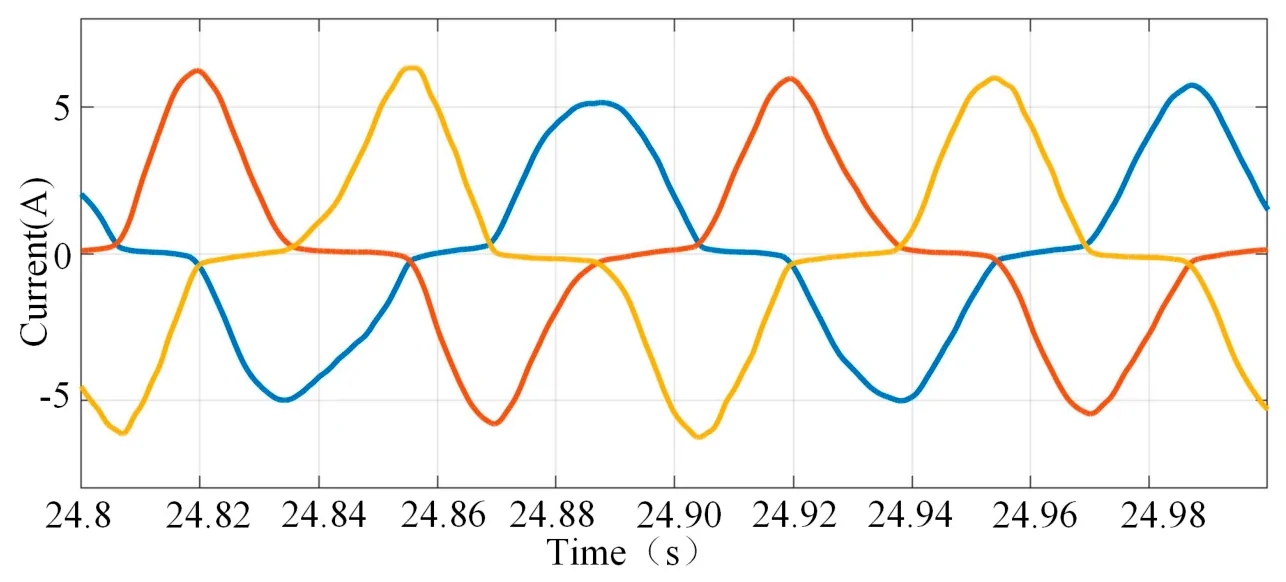
Three-phase currents with conventional dual-SVPWM.

Figure 5 illustrates the three-phase currents obtained using the conventional dual-SVPWM modulation strategy, where the colored curves represent the A-, B-, and C-phase currents, respectively, the same descriptions are also applied to the following figures. Since the dead-time voltage error is not considered in this algorithm, current distortion occurs whenever the phase current crosses zero. For a fair comparison, dead-time voltage compensation is not applied in any of the three experimental cases.

Because the phase angle between the voltage vectors generated by the two inverters is 120°, the common-mode voltages on both sides are theoretically identical. As a result, zero-sequence currents are not induced by the modulation strategy. This modulation scheme therefore ensures the minimum harmonic content.

**Figure 6:**
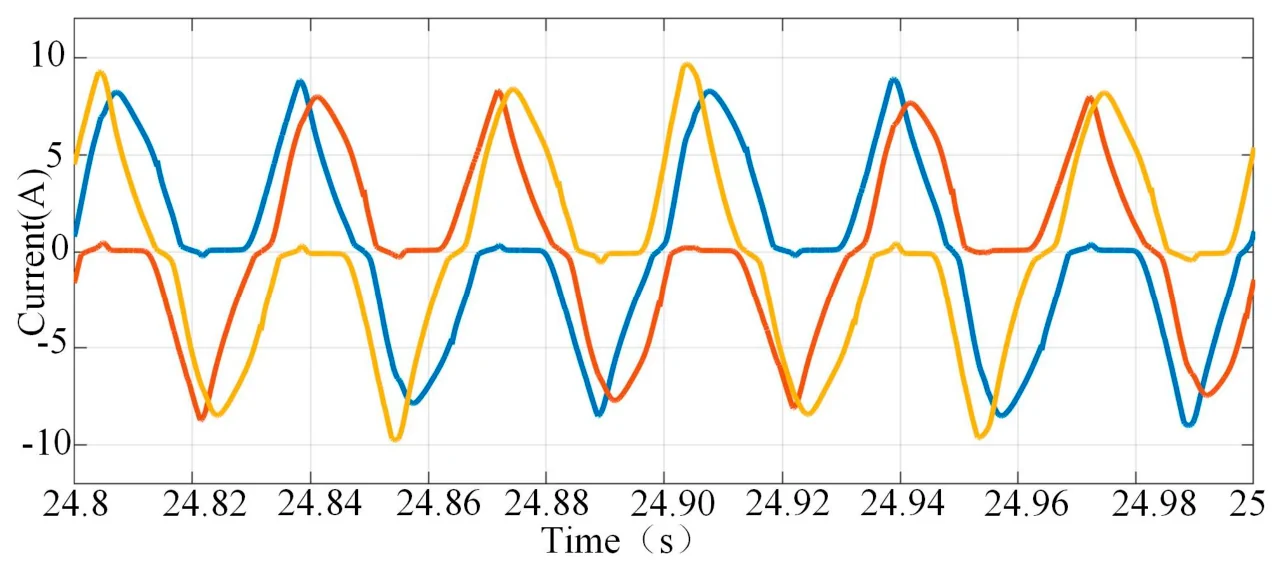
Three-phase currents using ASHCPWM without zero-sequence compensation.

Figure 6 shows the three-phase currents obtained using ASHCPWM without zero-sequence compensation. Since fixed switching combinations are alternately applied to the two inverters, the common-mode voltage inevitably introduces zero-sequence current harmonics if no common-mode voltage compensation is implemented. Consequently, the three-phase currents contain a large zero-sequence component and exhibit severe waveform distortion. Therefore, the current waveforms in Figure 6 differ significantly from the conventional balanced three-phase sinusoidal currents. This phenomenon is an inherent consequence of the common-mode voltage mismatch in the uncompensated ASHCPWM scheme rather than an abnormal experimental result.

In addition, because the dead-time voltage error is not considered in the algorithm, the harmonics contained in the three-phase currents include both those introduced by the modulation strategy and those caused by the dead-time effect.

**Figure 7:**
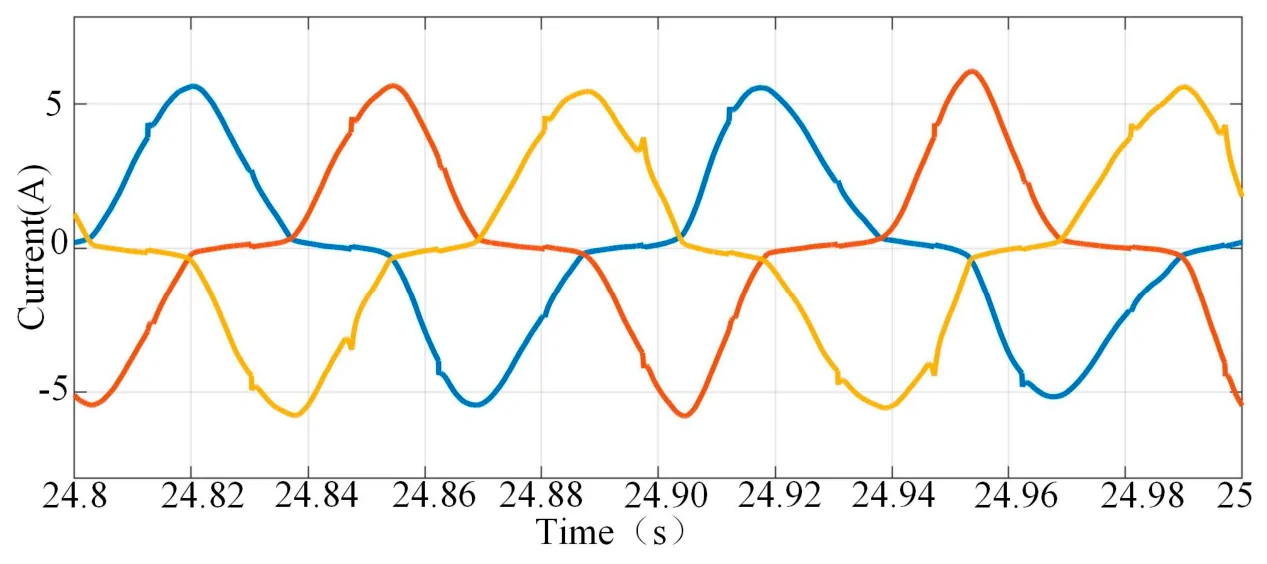
Three-phase currents using ASHCPWM with zero-sequence compensation.

Figure 7 illustrates the three-phase currents corresponding to the ASHCPWM with zero-sequence voltage compensation. By actively injecting a zero-sequence voltage component to counteract the zero-sequence voltage fluctuations introduced by the modulation strategy, the zero-sequence current is effectively suppressed. In addition, since the dead-time voltage error is not considered in this algorithm, distortion occurs in the three-phase currents around the zero-crossing points. However, compared with the ASHCPWM without zero-sequence voltage compensation, the current distortion is significantly reduced.

**Figure 8:**
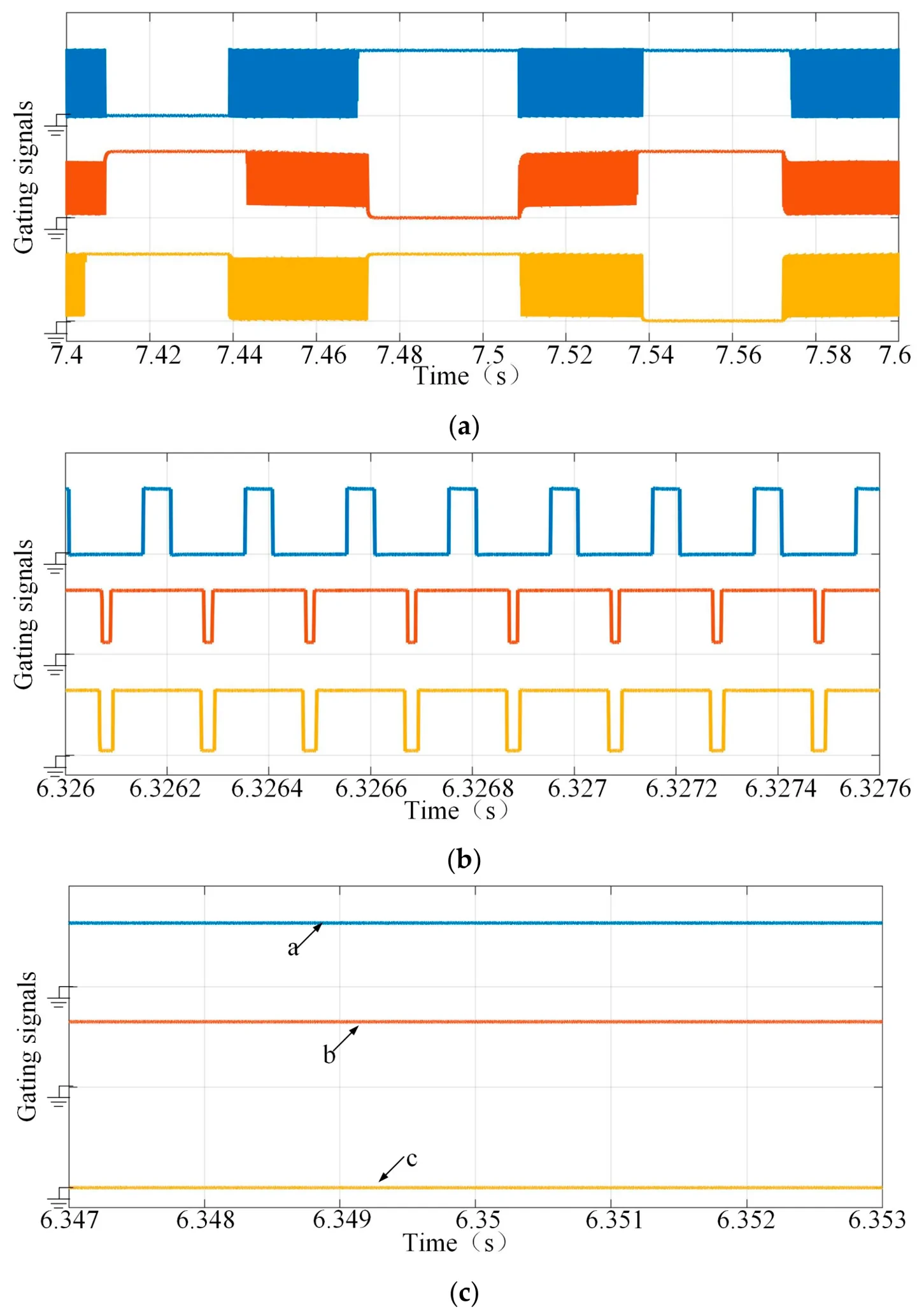
Drive signals of upper IGBTs in Inverter I. (**a**) Drive signals of Inverter I. (**b**) Waveform during SVPWM operation. (**c**) Waveform of fixed drive signals.

When ASHCPWM is employed, the three-phase gating signals of Inverter I are shown in Figure 8. In Sectors I, III, and V, Inverter II modulates the voltage vectors using the SVPWM scheme, while the three-phase gating signals of Inverter I remain unchanged. In Sectors II, IV, and VI, Inverter I modulates the voltage vectors using SVPWM, whereas the three-phase gating signals of Inverter II remain unchanged. In Figure 8c, a, b, and c represent the fixed gate signals of the phase-a, -b, and -c upper IGBTs of Inverter I, respectively.

**Figure 9:**
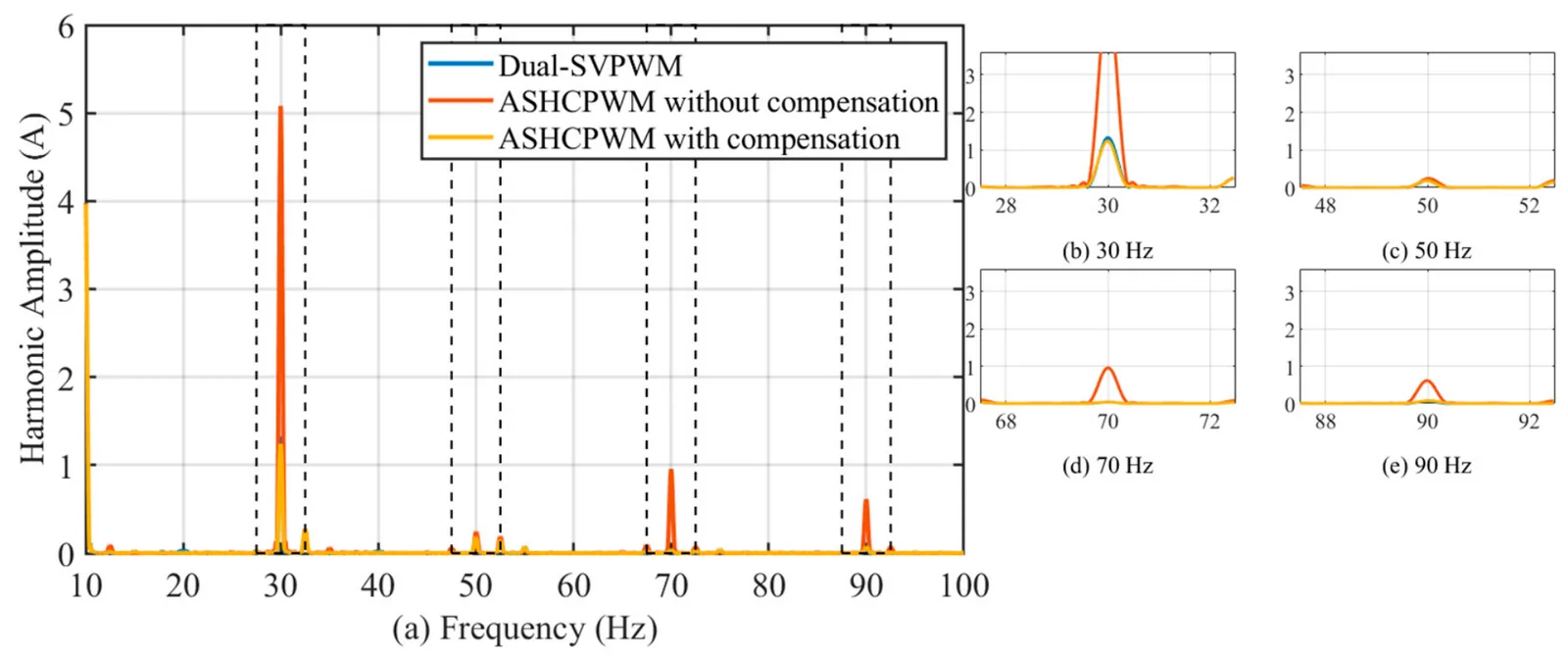
(**a**) Phase current harmonic comparison between 3 modulations: (**b**) 3rd-order harmonics; (**c**) 5th-order harmonics; (**d**) 7th-order harmonics; (**e**) 9th-order harmonics.

The comparison of the harmonic magnitudes of the three-phase currents obtained from the above three experiments is shown in Figure 9. Since none of the three experiments employs dead-time compensation, the harmonic comparison mainly reflects the modulation-induced harmonic currents. When the switching frequency is not considered and the conventional dual-SVPWM modulation scheme is adopted, the harmonic magnitudes are the smallest, consisting only of the third, fifth, and other harmonics caused by the dead time. With ASHCPWM without zero-sequence voltage compensation, the harmonic magnitudes increase significantly. In contrast, when ASHCPWM with zero-sequence voltage compensation is applied, the harmonic magnitudes are substantially reduced and are almost indistinguishable from those obtained with the conventional dual-SVPWM modulation. This indicates that the proposed ASHCP’WM with zero-sequence voltage compensation can suppress the zero-sequence current to a level comparable to that of the conventional method while retaining the reduced-switching characteristic of ASHCPWM.

The above comparison further demonstrates the advantage of the proposed modulation strategy. Compared with the conventional ASHCPWM without zero-sequence compensation, the proposed method significantly reduces the modulation-induced zero-sequence current and improves the phase-current quality. Compared with the conventional dual-SVPWM, the proposed method achieves a comparable level of harmonic suppression while retaining the characteristic that one inverter is clamped during the corresponding operating interval, thereby avoiding unnecessary simultaneous switching actions of the two inverters. Therefore, the proposed strategy provides a favorable compromise between current quality and the number of switching actions in the IGBT-based OEW-PMSM drive.

### 4.2. Harmonic Suppression Comparison

Although the proposed ASHCPWM method can effectively suppress zero-sequence current harmonics, the three-phase currents still contain considerable harmonic components due to the effects of dead-time voltage errors and other nonideal factors. Therefore, on the basis of the proposed modulation strategy, improved harmonic suppression methods are required to further reduce the current harmonics. In the experiments, harmonic extraction is performed using the conventional multiple rotating reference frame method combined with low-pass filters, the traditional Kalman filter-based harmonic extraction approach, and an improved Kalman filter-based method, and the performance of these methods is comparatively evaluated.

First, the conventional multiple rotating reference frame combined with a low-pass filter (LPF) is adopted to extract the positive-sequence fundamental component and the negative-sequence fifth-order harmonic from the three-phase stator currents, while the zero-sequence third-order harmonic is not considered due to the limitation of this method.

**Figure 10:**
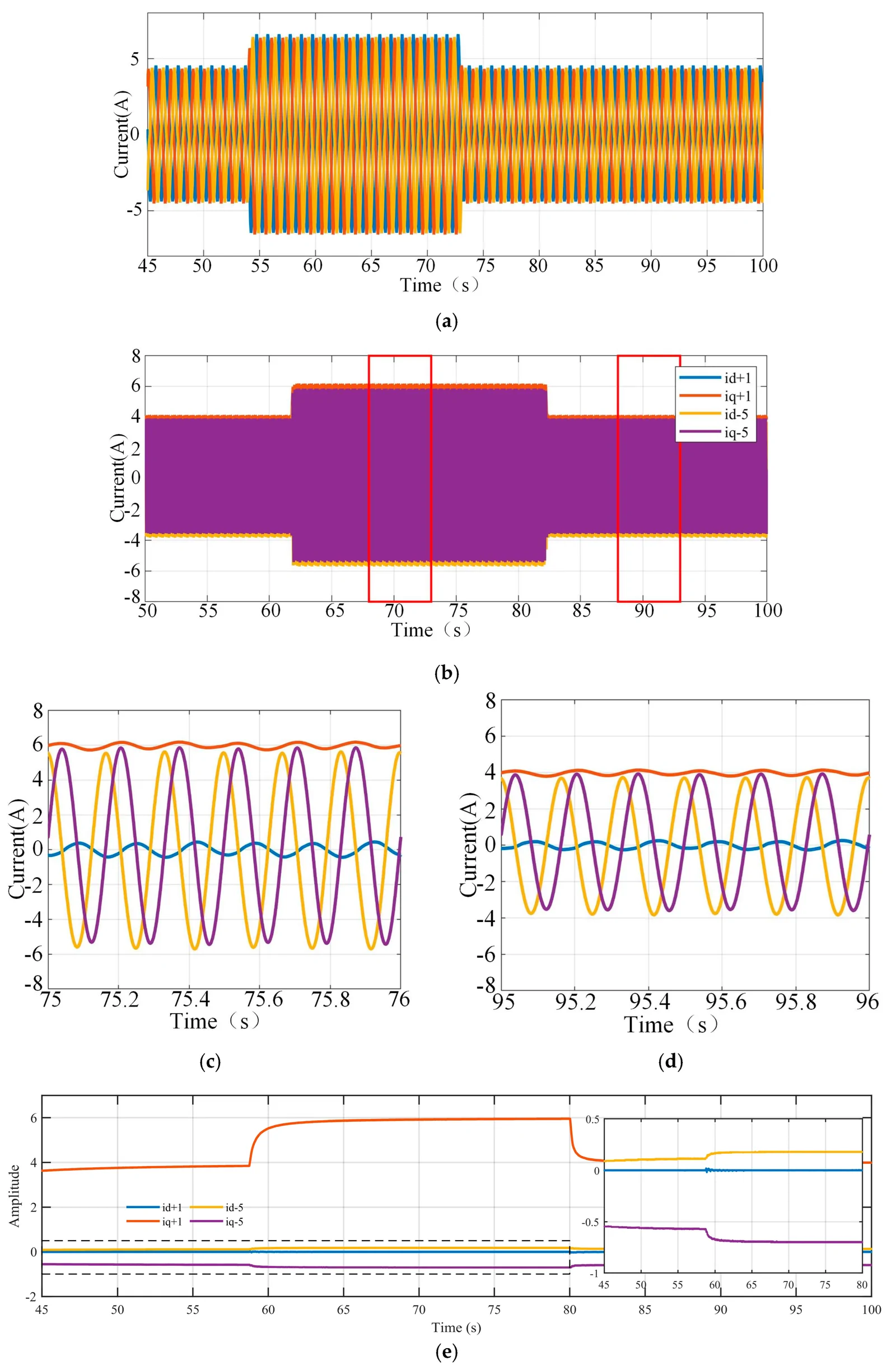
Conventional multiple rotating reference frame combined with a low-pass filter. (**a**) Three-phase currents; (**b**) extracted fundamental and 5th-order harmonic in dq-axes; (**c**) 75–76 s; (**d**) 95–96 s; (**e**) extracted fundamental and 5th-order harmonic with LPF in dq-axes.

**Figure 11:**
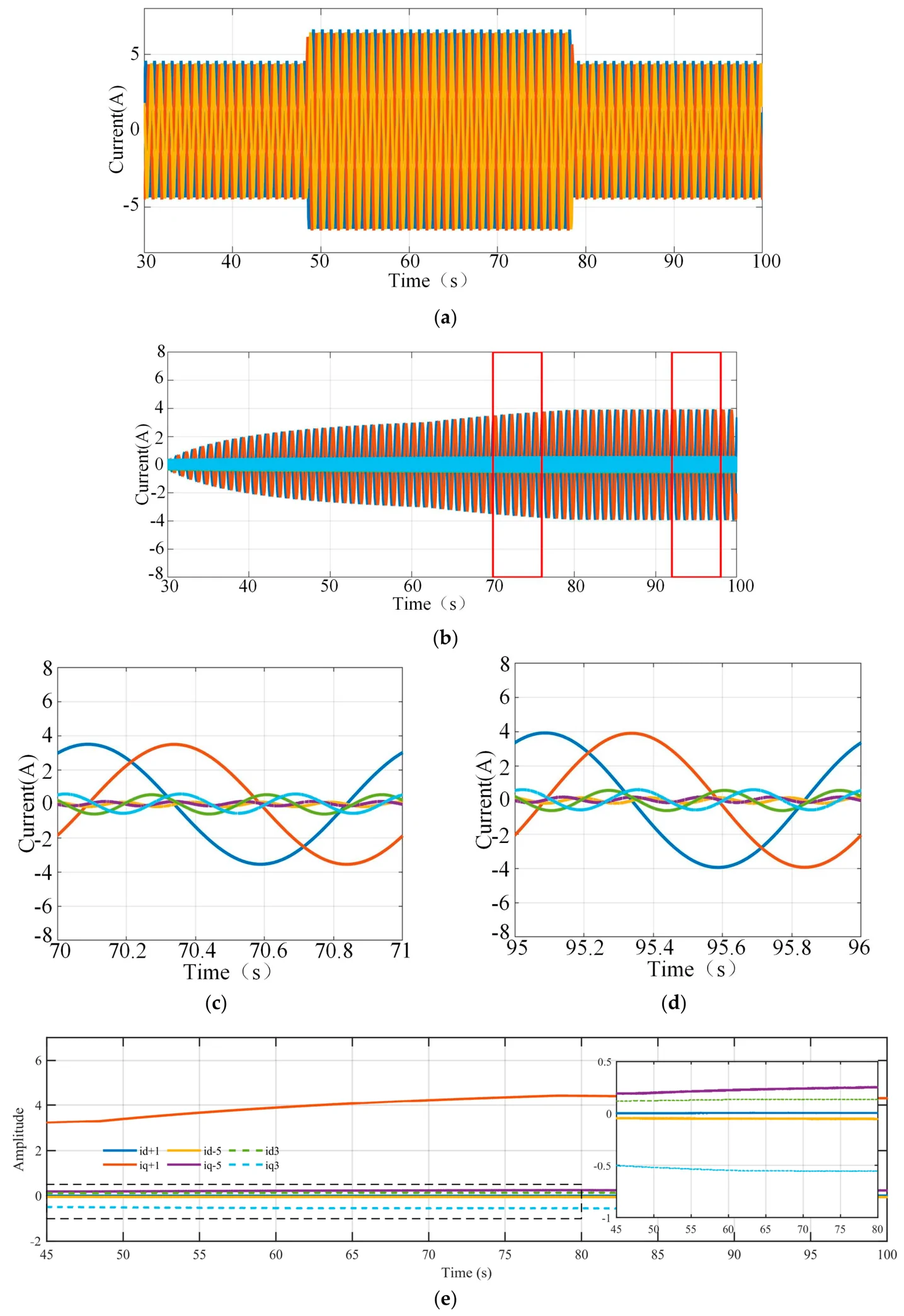
Conventional Kalman filter-based harmonic extraction method. (**a**) Three-phase currents; (**b**) extracted harmonics with conventional Kalman filter in αβ-axes; (**c**) 75–76 s; (**d**) 95–96 s; (**e**) extracted harmonics with conventional Kalman filter in dq-axes.

**Figure 12:**
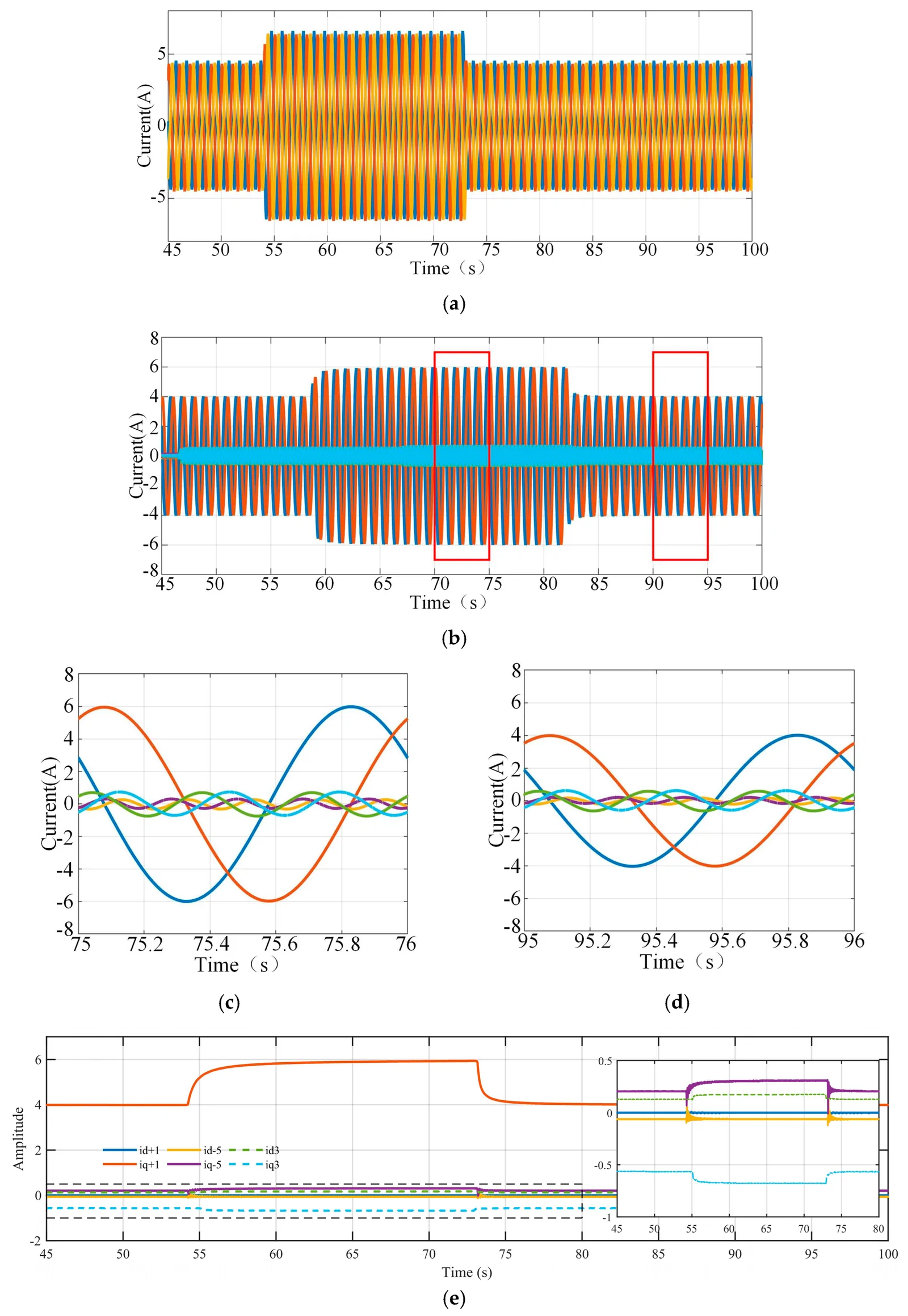
Improved Kalman filter-based harmonic extraction method. (**a**) Three-phase currents; (**b**) extracted harmonics with improved Kalman filter in αβ-axes; (**c**) 75–76 s; (**d**) 95–96 s; (**e**) extracted harmonics with improved Kalman filter in dq-axes.

To independently evaluate the harmonic extraction performance of the proposed method, the rotor position of the PMSM was fixed during the experiments shown in Figure 10, Figure 11 and Figure 12. The fundamental and harmonic current components were actively injected with prescribed reference amplitudes, and the corresponding components extracted by different methods were compared. Therefore, these tests do not represent the normal rotating operating condition of the PMSM. Here, idref+1 and idref+3 denote the d-axis reference amplitudes of the fundamental (first-order) current component and the third-order harmonic current component, respectively. Identical reference amplitudes were intentionally assigned to facilitate a direct comparison of the tracking and extraction performance of the proposed algorithm, rather than to represent an actual operating condition of the motor. For consistency among the comparative experiments, the electrical frequency was fixed at 10 Hz.

The measured three-phase stator currents are shown in Figure 10a. The extracted results in the two-phase stationary reference frame are illustrated in Figure 10b, where it can be observed that the amplitudes of the harmonic components vary slowly during step changes because of the limited bandwidth of the LPF. The transient intervals highlighted by the red boxes in Figure 10b are enlarged in Figure 10c and Figure 10d, respectively, to provide a clearer view of the harmonic extraction dynamics. Finally, after transforming each component into its corresponding rotating reference frame, the amplitudes of the fundamental and fifth-order harmonic are obtained with LPF, as shown in Figure 10e.

As shown in Figure 10b, since the fundamental frequency is the dominant component of the signal while the fifth harmonic has a relatively small amplitude, the extracted fundamental-frequency dq components contain less harmonic interference from the fifth harmonic, whereas the extracted fifth-harmonic dq components are more significantly affected by the fundamental-frequency harmonic interference. The ultimate goal of signal extraction is to completely eliminate the AC components in each component and retain only the DC part. If a lower filter bandwidth is chosen, the AC components can be effectively rejected, but the bandwidth for DC extraction becomes too low, resulting in an overly slow dynamic response. Conversely, if a higher filter bandwidth is chosen, a faster dynamic response can be achieved, but the extracted signal will fail to eliminate the interference from AC components. Therefore, a trade-off must be made between dynamic response speed and steady-state accuracy.

The experimental results of the conventional Kalman filter-based harmonic extraction method are presented in Figure 11. Figure 11a shows the three-phase current waveforms under harmonic magnitude variations. The corresponding harmonic extraction results are illustrated in Figure 11b–e. After the transient process, the positive-sequence fundamental component, the negative-sequence fifth-order harmonic, and the zero-sequence third-order harmonic are accurately extracted with little mutual interference. However, the responses around the magnitude variations in Figure 11c,d indicate a relatively slow convergence to the new steady-state values. This limitation arises because the error covariance of the conventional Kalman filter gradually converges to a steady-state value, leading to a reduced Kalman gain. Consequently, the estimator becomes less responsive to abrupt harmonic variations, resulting in unsatisfactory dynamic tracking performance.

An improved Kalman filter-based harmonic extraction method is developed by adaptively adjusting the a posteriori error covariance according to the harmonic extraction error. The three-phase current waveforms, the extracted positive-sequence fundamental current, the negative-sequence fifth-order harmonic, and the zero-sequence third-order harmonic are shown in Figure 12. It can be observed that, after the harmonic extraction reaches steady state, further variations in the harmonic magnitudes are tracked with a significantly enhanced dynamic response, while the extracted harmonics remain free from interference by other harmonic components in steady state. Compared with the conventional multiple rotating reference frame method combined with low-pass filters, the proposed improved Kalman filter-based harmonic extraction approach effectively improves the dynamic response while maintaining steady-state accuracy, resulting in a substantial enhancement in harmonic extraction performance.

These experimental results further demonstrate that the proposed improved Kalman filter achieves faster transient harmonic tracking than both the conventional LPF-based method and the conventional Kalman filter, while maintaining high steady-state extraction accuracy.

**Figure 13:**
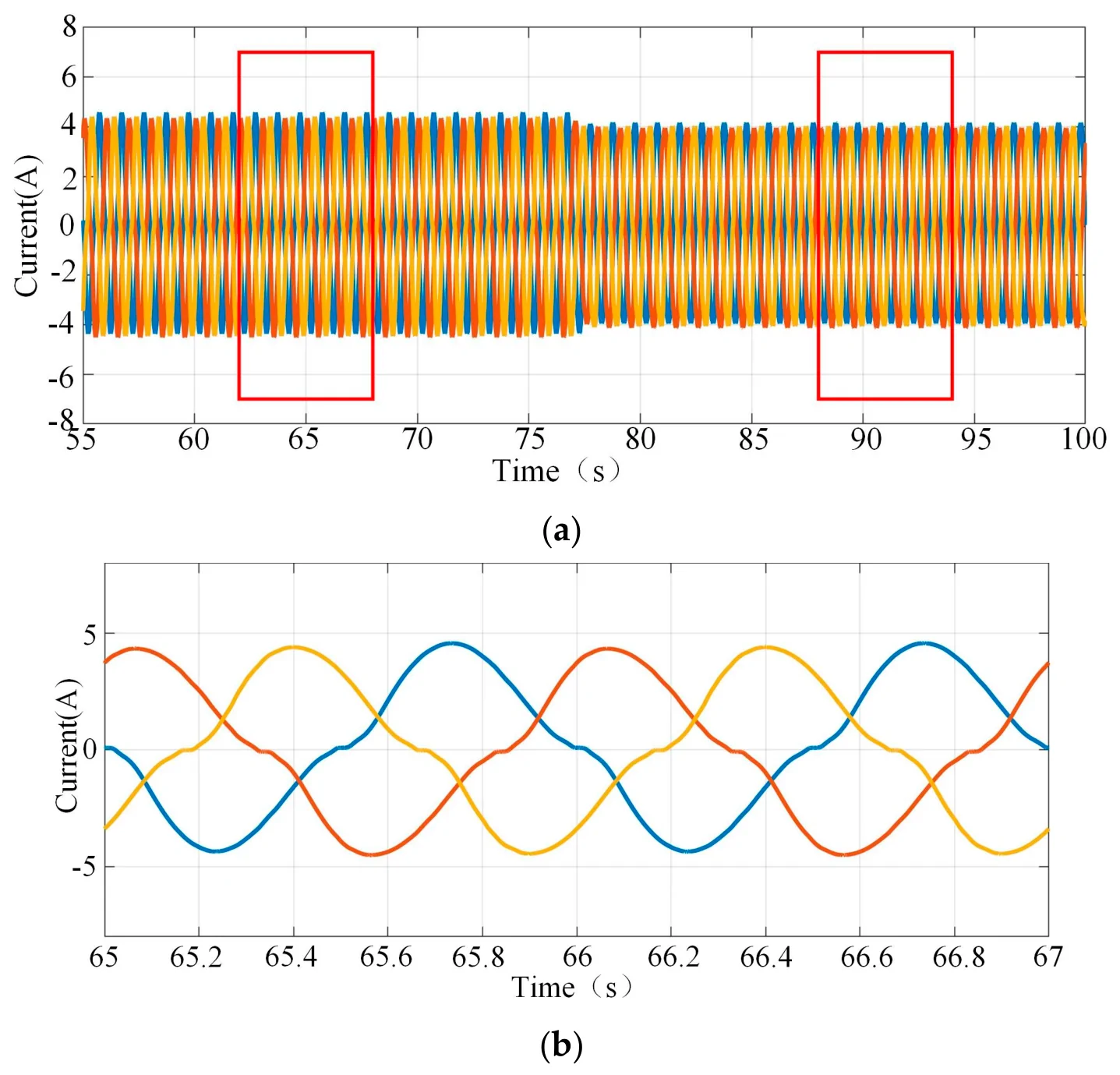

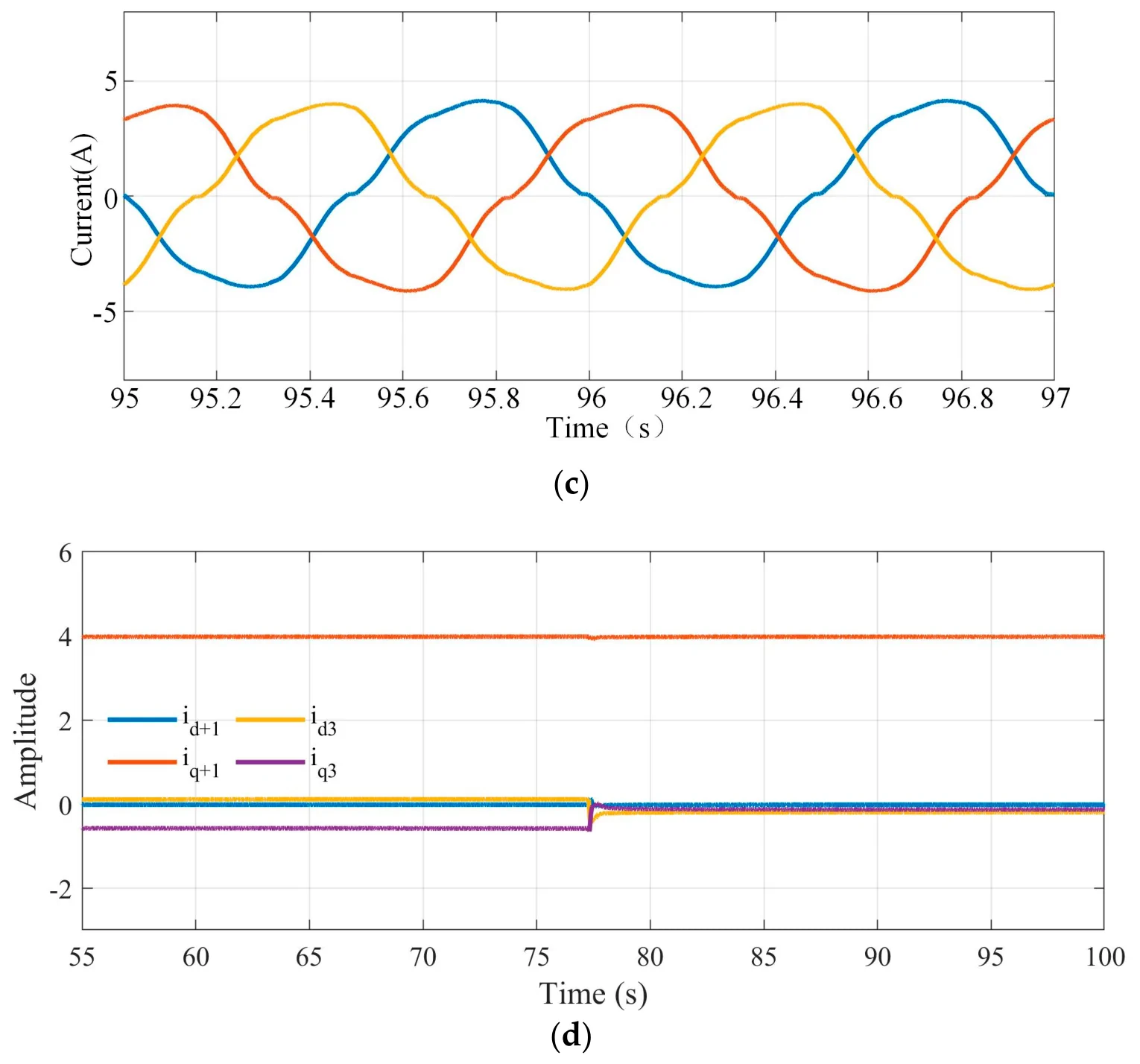
Comparison between phase currents without harmonic suppression and with improved Kalman filter-based harmonic extraction method. (**a**) Three-phase currents. (**b**) Three-phase currents without harmonic suppression. (**c**) Three-phase currents with improved Kalman filter-based harmonic extraction method. (**d**) Positive- and negative-sequence 3rd-order dq-axes waveforms.

As shown in Figure 13, the current waveforms during the transition from the conventional control scheme without harmonic closed-loop control to the improved Kalman filter-based harmonic extraction method are presented, where the reference current amplitude is set to 4A. The transient intervals highlighted by the red boxes in Figure 13a are enlarged in Figure 13b and Figure 13c, respectively, to provide a clearer view of the phase currents. Prior to the switching instant, distortion occurs around the zero-crossing points of the three-phase currents due to the dead-time effect, which leads to overshoot in the integral term of the PI controller and consequently causes current overshoot at the peak values. After applying the improved Kalman filter-based harmonic extraction method, the dominant third-order harmonic component in the three-phase currents is effectively suppressed. As a result, the current amplitude no longer exhibits overshoot at the peaks and is accurately constrained to 4A. Owing to the high dynamic response capability of the improved Kalman filter-based harmonic extraction method, the closed-loop control of the third-order harmonic also achieves a fast transient response.

**Figure 14:**
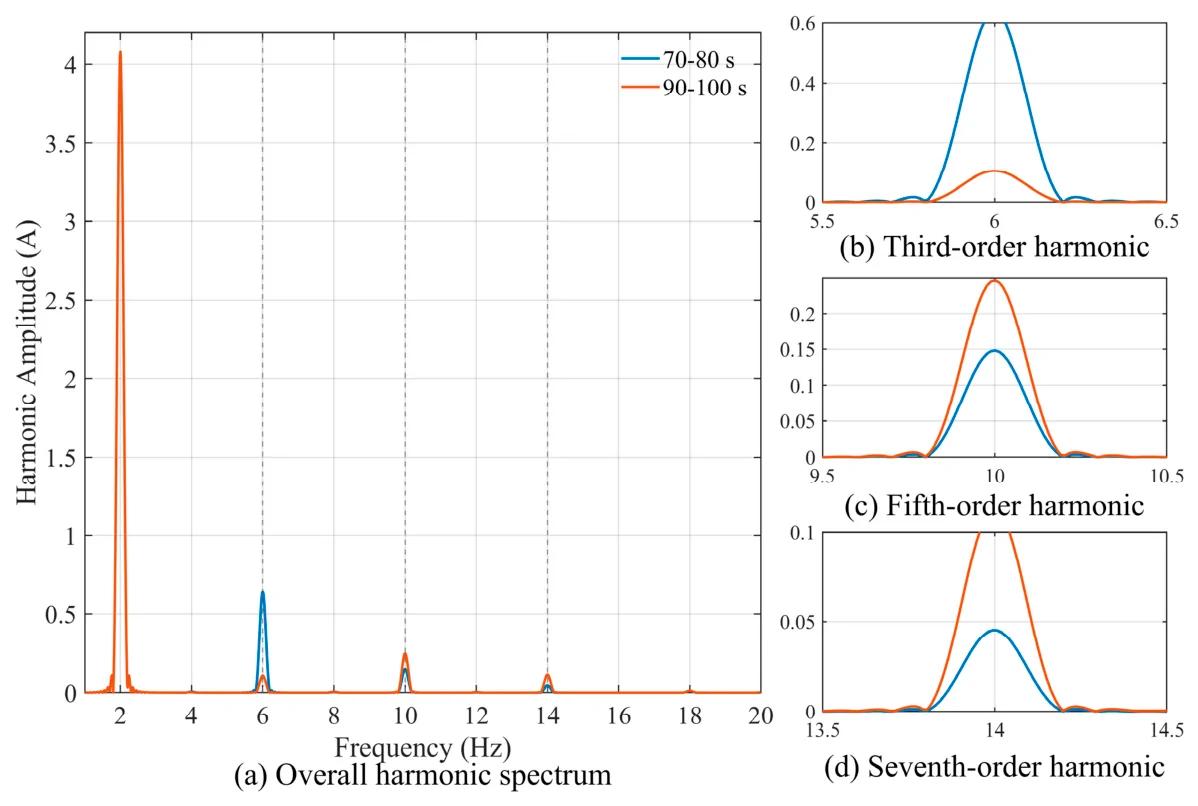
Overall harmonic spectrum and partial enlarged details. (**a**) Overall harmonic spectrum; (**b**) 3rd-order harmonic; (**c**) 5th-order harmonic; (**d**) 7th-order harmonic.

A comparison of the current harmonic spectra with and without the harmonic closed-loop control based on the improved Kalman filter-based harmonic extraction method is illustrated in Figure 14. Since only the third-order harmonic is extracted and regulated through closed-loop control, its amplitude is significantly suppressed. In contrast, because no closed-loop control is implemented for the negative-sequence fifth-order harmonic, its amplitude remains nearly unchanged.

The experimental results demonstrate that, at different operating speeds, the proposed improved Kalman filter-based harmonic extraction method can rapidly extract the fundamental and third-order harmonic amplitudes, and the harmonics induced by the dead-time effect are effectively mitigated.

## 5. Conclusions

This paper proposes an improved ASHCPWM-based control strategy for common DC-bus OEW-PMSM drives to reduce IGBT switching actions while suppressing zero-sequence and harmonic currents. By alternately clamping one inverter while the other performs PWM modulation, the proposed modulation strategy reduces the number of switching transitions and the effective switching frequency of the IGBTs compared with the conventional dual-SVPWM strategy. Meanwhile, the common-mode voltage characteristics of ASHCPWM are analyzed, and a zero-sequence voltage compensation method is introduced to mitigate the zero-sequence current caused by the common-mode voltage mismatch between the two inverters. Experimental results demonstrate that the proposed method effectively suppresses the modulation-induced zero-sequence harmonics while retaining the reduced-switching characteristic of ASHCPWM.

Furthermore, an improved Kalman filter-based harmonic extraction and closed-loop suppression method is developed to address the harmonic currents caused by dead-time effects and other nonideal factors. By incorporating mathematical models of the fundamental and harmonic components, the proposed method reduces mutual interference among different harmonic components and maintains high steady-state extraction accuracy. In addition, the error covariance is adaptively adjusted according to the observation error, thereby improving the dynamic tracking capability when harmonic amplitudes vary. The experimental results verify that the proposed method provides a faster dynamic response than the conventional harmonic extraction methods while maintaining satisfactory steady-state accuracy. If additional harmonic components need to be considered, the state dimension of the improved Kalman filter can be further extended to enable the extraction and closed-loop regulation of a wider range of harmonics.

## Figures and Tables

**Table 1:** Main specifications of the reference PMSM.

Parameter	Symbol	Value
Rated power	*P*_N_	750 W
Rated voltage	*U*_N_	200 V
Rated current	*I*_N_	4.1 A (rms)
Rated torque	*T*_N_	2.39 N·m
Maximum torque	*T*_max_	7.16 N·m
Rated speed	*n*_N_	3000 r/min

## Data Availability

The original contributions presented in this study are included in the article. Further inquiries can be directed to the corresponding authors.
